# Relative importance of the EQ-5D five dimensions among patients with chronic diseases in South Korea – a comparison with the general population preference weights

**DOI:** 10.1186/s12955-018-0987-7

**Published:** 2018-08-03

**Authors:** Jihyung Hong

**Affiliations:** Department of Healthcare Management, Gachon University, Seongnam, 13120 South Korea

**Keywords:** Patient preference, Quality of life, EQ-5D; VAS

## Abstract

**Background:**

Who should provide the values of health states in economic evaluations of health technologies has long been the subject of debate. This study examined and compared the relative importance of the EQ-5D five dimensions, using both patient-reported values and general population tariffs, among patients with chronic diseases in South Korea and also assessed the pattern of the discrepancy between patient and general population values by type of chronic diseases.

**Methods:**

Data were taken from the 2013 Korea Health Panel survey. This analysis focused on adult patients with chronic diseases (*n* = 3216). Patient-reported EQ-5D profiles and visual analogue scale (VAS) values were used to assess the relative importance of the EQ-5D five dimensions among these patients, using a linear regression model. The relative importance of the EQ-5D dimensions was then compared to the EQ-5D tariffs elicited from the general population. The relative magnitude of the discrepancies between patient and general population values was also assessed by type of chronic diseases.

**Results:**

Anxiety/depression and pain/discomfort appeared to have the largest impact on the self-rated patient VAS, which fairly contrasted with the general population model. In addition, a further regression analysis showed that the discrepancy between patient and general population values varied with the type of chronic diseases. The greatest discrepancy between the two was found in patients with diseases of the blood and blood-forming organs, neoplasms and diseases of the digestive system.

**Conclusions:**

These analyses revealed differences in the relative weights attached to the EQ-5D dimensions between patient groups and the general population, particularly in those ‘non-tangible’ dimensions. These differences consequently led to greater discrepancies between patient and general population values in certain patient groups, which can have significant implications for resource allocation decisions in South Korea.

## Background

Economic evaluations can provide ‘value-for-money’ information to decision makers who need to allocate limited healthcare resources as efficiently as possible. Over the past decades, an increasing number of countries have formally/informally adopted an economic evaluation as an aid for such decision making. In Asia, South Korea has been the first country to officially require and assess economic evaluation data for drug reimbursement decision making, which was introduced as a part of the Drug Expenditure Rationalization Plan implemented in December 2006 [1, 2]. New drugs that have failed to prove their cost-effectiveness, that is, their incremental costs per incremental quality-adjusted life years (QALYs) are greater than per capita GDP, are now unlikely to be recommended in South Korea [3]. There are, however, a lot of issues in measuring costs and QALYs, which can have significant implications for resource allocation decisions. One of those critical issues would be whose preferences should count when valuing health states and thereby calculating QALYs, which has long been the subject of debate [4, 5].

The QALY combines both quality and quantity of life into a single metric. The quality of life (QoL) component requires the valuation of being in a particular health state. This can be done by asking patients who are actually experiencing the health state. Another approach is to ask members of the general population to imagine being in the health state. Generic preference-based measures such as EQ-5D [6] and SF-6D [7] are commonly used in this type of valuation, which define the health states in terms of generic dimensions of health, for instance, mobility, self-care, usual activities, pain/discomfort and anxiety/depression dimensions in the case of the EQ-5D. The South Korean pharmaco-economic evaluation guideline [8] states that the general population is preferred as a source of preference, which is consistent with the recommendation made by the Washington Panel on Cost-effectiveness in Health and Medicine [9] and many other countries [10, 11]. The most popular arguments for the use of the general population preferences are that community members can value health states hidden behind a ‘veil of ignorance’, making them blind to their own self-interest [12]. They can also better represent the view of taxpayers and potential patients. In addition, patients can adapt to their health states, which could make them value their health states higher than the general population and consequently lead to a lower priority for their treatments [13–18]. Despite these reasons, there are also economic guidelines that recommend or advocate the use of patient values. For instance, in Sweden and Denmark, patient preferences are preferred because they are the ones best informed about being in those health states [10]. This view is partly supported by a recent proof-of-concept study [19], which shows that members of the public may not be fully aware of the consequences of ill health.

Empirical evidence, however, shows mixed results on the discrepancies between patient and general population preferences. A meta-analysis by Dolders et al. [20], using 78 estimators from 33 studies with various patient groups and valuation techniques, shows no significant differences between patient preferences for actual health states and general population preferences for hypothetical health states. Meanwhile, a more recent meta-analysis by Peeters et al. [17], using 40 estimators from 30 studies, suggests that patients tend to value their health states higher than members of the general public. Notably, most of the previous studies are based on small sample sizes and do not provide information on which particular dimensions of health patients would consider more important or less important compared to the general population. More recently, some studies have attempted to employ larger sample sizes to compare patient and general population preferences across multiple patient samples or the general population, using the EQ-5D [21]. For instance, Mann et al. [22], with 3376 UK patients, examined discrepancies in the relative importance of the five EQ-5D dimensions between patients and the general public, using patient visual analogue scale (VAS) values and general population VAS tariffs. They found that patients tend to consider anxiety/depression more important than the general population. Similar findings were also reported by Burström et al. [23] with a Swedish sample (*n* = 45,000) and Sun et al. [24] with a Chinese sample (*n* = 120,709), using experience-based VAS values. Meanwhile, Rand-Hendriksen et al. [25] reported that usual activities are likely the most important dimensions among the US patients, whereas self-care and pain/discomfort are likely the most important dimensions among the US general population. Taken together, this recent evidence suggests that discrepancies do exist between patient and general population preferences across the countries but what dimensions of health are considered more important among patients, relative to the general public, may differ across cultures.

In South Korea, three EQ-5D-3 L valuation studies are publicly available, which provide the time trade-off (TTO) valuation algorithms that can be used to convert the EQ-5D profile data into a single numeric EQ-5D index score [26–28]. The valuation algorithms developed by Lee et al. [26] are most commonly used in South Korea (e.g., [29]) due to the representativeness of its sample. They have developed the algorithms based on the valuation of 101 EQ-5D health states with a South Korean representative sample of 1307 respondents, who were selected through a multistage stratified random sampling. These valuation algorithms indicate that the general population tend to consider health problems less problematic than their UK counterparts [8, 30], especially in anxiety/depression and pain/discomforts dimensions. A similar pattern has also been observed in the Japanese EQ-5D-3 L valuation study [31]. Feng et al. [32] have postulated that the general population in Japan, possibly in South Korea as well, are less willing to trade quantity with quality of life, particularly in ‘non-tangible’ dimensions that the general population may have some difficulties to imagine. It would be of great interest whether patients in South Korea also have similar attitudes or preferences towards the EQ-5D dimensions.

This study thus aimed to assess and compare the relative importance of the EQ-5D five dimensions among patients with chronic diseases in South Korea, using both patient-reported VAS values and general population tariffs applied to patient-reported EQ-5D profiles taken from a nationally representative survey. The study also assessed the pattern of the discrepancy between patient and general population values by type of chronic diseases to draw resource allocation implications of using a different source of preference. These empirical findings may facilitate an in-depth discussion of the issues relating to the question of whose preferences should count, which have important implications for resource allocation decisions such as reimbursement and priority setting in South Korea and possibly in other Asian countries that refer to the Korean model. Nevertheless, it should be made clear that these findings represent only the relative importance of the EQ-5D dimensions given by South Korean patients and general population and therefore cannot be generalised to other countries.

## Methods

### Data and study population

Data for this study were taken from the 2008–2014 Korea Health Panel (KHP) survey (beta version 1.3), which is a nationally representative panel survey of health-related outcomes and other data elements conducted by the Korea Institute for Health and Social Affairs and the National Health Insurance Service in South Korea [29]. Participants were selected from non-institutionalized civilians through a stratified multistage probability sampling design. Data on socio-demographic characteristics and health-related outcomes including EQ-VAS values and EQ-5D-3 L profiles were collected through face-to-face interviews. This study used the data collected in 2013 (14,839 individuals from 5200 households), which was the final year in which EQ-5D data were collected.

### Study sample

This study included those participants who (1) were at least 18 years old, (2) reported having had doctor-diagnosed chronic diseases in the past 1 year, and (3) reported at least some problems in one of the EQ-5D dimensions. Chronic diseases were coded with the Korean Standard Classification of Diseases 6th edition (KCD-6) in the KHP dataset [33], and the participants were categorised into 15 groups by type of chronic diseases.

### EQ-5D and EQ-VAS

The EQ-5D is a self-reported generic preference-based measure of health, developed by the EuroQol Group [6]. It has two elements, which are the EQ-5D descriptive system and the EQ-VAS. The EQ-5D descriptive system describes health in terms of five dimensions: mobility, self-care, usual activities, pain/discomfort and anxiety/depression. Each dimension can be rated at three levels (EQ-5D-3 L), corresponding to no problems (level 1), some or moderate problems (level 2) and severe problems (level 3). These five dimensions and levels define a total of 243 (3^5^) health states, which can also be described in the form of a five-digit number describing the levels for each dimension (e.g., 12332). Each EQ-5D health state can then be scored using the valuation algorithms developed for such a purpose – this study used the one developed by Lee et al. with data from the South Korean general population [26]. The EQ-VAS is a visual analogue scale, which asks respondents to rank their current health states from ‘best (100)’ to ‘worst (0)’.

### Statistical analysis

Of the 11,999 individuals with the age of at least 18 years, 7387 reported having had a doctor-diagnosed chronic disease. Of these, 602 patients had incomplete data on the EQ-5D profiles and EQ-VAS and therefore were excluded from this analysis. Of the remaining 6785 patients, 3216 reported ‘some problems’ or ‘severe problems’ at least in one of the EQ-5D five dimensions – this analysis included these patients as they were the ones who were *experiencing* health problems at least in one of the EQ-5D health dimensions. Nevertheless, those participants reporting ‘no problems’ in all dimensions were also included in a sensitivity analysis.

The present study first described the sample characteristics and self-reported EQ-5D profiles using descriptive statistics (mean, standard deviation [SD] and %). Three main analyses were then conducted to assess whether and how patients’ health state valuations differ from the general population’s valuation.

First, it described the mean EQ-VAS values and the mean EQ-5D index scores calculated with general population EQ-5D tariffs by type of chronic diseases. The UK and Japanese tariffs were also used for a comparison purpose [31].

Second, an ordinary least squares (OLS) regression, as in the original EQ-5D valuation study by Lee et al. [26], was conducted to examine the impact of each EQ-5D dimension and level on patient VAS values. The following model was considered:

$$ 1-V=f\left({MO}_i,{SC}_i,{UA}_i,{PD}_i,{AD}_i,N3, controls\right). $$

Where V was the VAS value (divided by 100) of a patient’s health state and MO, SC, UA, PD and AD refer to each of the EQ-5D five dimensions. *i* is the level of each dimension, thus taking a value of 1, 2, or 3. N3 is a dummy variable indicating whether a respondent had level 3 at least in one of the five EQ-5D dimensions. The model was assessed (1) without any controls, (2) with demographic controls (age and sex), and (3) with socio-demographic controls (age, sex, marital status, living alone, educational attainment, employment status, medical aid, income quintiles and perceived social status as measured by the MacArthur Scale [34]).

Finally, a further regression analysis was conducted to examine the pattern of the discrepancies between patient and general population values by type of chronic diseases. The discrepancy between the two was included as a dependent variable. The following independent variables were included in the model: (1) dummy variables indicating whether or not a patient had each type of chronic diseases and (2) socio-demographic controls (age, sex, marital status, living alone, educational attainment, employment status, medical aid, income quintiles and perceived social status). The post-estimation was followed to estimate the adjusted mean differences between patient and general population values in each patient subgroup by type of chronic diseases. The adjusted mean differences were estimated at the mean level of each socio-demographic variable and at zero values of all other chronic disease dummy variables. Given the use of different preference elicitation method and anchoring, however, these mean differences should only be interpreted as the relative differences - not the absolute differences - between patient and general population preferences by type of chronic disease.

All statistical analyses were performed using STATA/SE version 11 [35].

## Results

A total of 3216 patients with chronic diseases were included in the present analysis. The mean age of the sample was 63.5 years (SD = 13.8) and 35.3% were male (Table 1). The most common chronic disease reported was diseases of the musculoskeletal system and connective tissue (65.5%), followed by diseases of the circulatory system (50.5%), diseases of the digestive system (37.3%), and endocrine, nutritional and metabolic disease (33.6%).

**Table 1:** The characteristics of survey participants with chronic diseases (*n* = 3216)

	Mean (SD) or %
Age, mean (SD)	63.5 (13.8)
18–45, %	11.5%
46–70, %	51.5%
≥ 71, %	37.0%
Male, %	35.3%
Marital status, %	
Married	70.7%
Single	3.7%
Widowed/Separated/divorced	25.6%
Living alone, %	15.2%
Level of education, %	
≤ Elementary	47.9%
Middle School	16.7%
High School	23.8%
≥ University	11.7%
Employment status, %	
Regular/long-term	25.0%
Non-regular/temporary	23.5%
Unemployed	2.5%
Economically inactive/students	50.0%
Medical aid, %	8.3%
Perceived social status	3.6 (1.5)

*Abbreviation: SD* standard deviation

Table 2 demonstrates the level of impairment in each of the EQ-5D five dimensions experienced by these patients. More than half of the patients reported ‘no problems’ in each of the EQ-5D dimensions, except for the pain/discomfort dimension. In the pain/discomfort dimension, the majority (81.3%) reported ‘moderate problems’. As for ‘severe health problems’, only a few patients reported this level of health problems in any of the dimensions. Conducting a further regression analysis was nevertheless possible because there were at least 30 patients reporting ‘severe health problems’ in each of the EQ-5D dimensions with being the lowest in the self-care dimension (*n* = 30, 0.9%) and being the highest in the pain/discomfort dimension (*n* = 150, 4.7%). The most frequently reported health state was 11121 (34.5%), followed by 11122 (10.1%), 21121 (8.9%), 11112 (8.0%), 21221 (5.4%), and 22221 (5.1%).

**Table 2:** Distribution of problems by the EQ-5D dimensions and levels among patients with chronic diseases (*n* = 3216)

	Mobility	Self-care	Usual activities	Pain/Discomfort	Anxiety/Depression
No problems (level 1)	1895 (58.9%)	2729 (84.9%)	2291 (71.2%)	453 (14.1%)	2035 (63.3%)
Moderate problems (level 2)	1286 (40.0%)	457 (14.2%)	882 (27.4%)	2613 (81.3%)	1130 (35.1%)
Severe problems (level 3)	35 (1.1%)	30 (0.9%)	43 (1.3%)	150 (4.7%)	51 (1.6%)

The mean patient VAS value was 0.61 (SD = 0.17), which was smaller than the mean EQ-5D index score calculated with the Korean general population tariffs (mean = 0.82, SD = 0.13) (Table 3). Notably, this mean EQ-5D index score was greater than those calculated with both the UK (mean = 0.70, SD = 0.19) and Japanese (mean = 0.69, SD = 0.11) general population tariffs. The mean patient VAS value and the mean EQ-5D index score remained largely similar across all types of chronic diseases.

**Table 3:** The mean level of patient VAS values and EQ-5D general population TTO values by type of chronic diseases

All	n	EQ-VAS	EQ-5D (KR)	EQ-5D (UK)	EQ-5D (JP)
	3216 (100.0%)	0.61 (0.17)	0.82 (0.13)	0.70 (0.19)	0.69 (0.11)
Certain infectious and parasitic diseases (A00-B99)	306 (9.5%)	0.61 (0.17)	0.81 (0.13)	0.68 (0.22)	0.69 (0.12)
Neoplasms (C00-D48)	308 (9.6%)	0.59 (0.18)	0.82 (0.15)	0.69 (0.22)	0.69 (0.12)
Diseases of the blood and blood-forming organs and certain disorders involving the immune mechanism (D50-D89)	57 (1.8%)	0.57 (0.20)	0.83 (0.11)	0.69 (0.20)	0.69 (0.10)
Endocrine, nutritional and metabolic diseases (E00-E90)	1079 (33.6%)	0.59 (0.17)	0.81 (0.13)	0.68 (0.21)	0.68 (0.12)
Mental and behavioural disorders (F00-F99)	267 (8.3%)	0.57 (0.18)	0.77 (0.18)	0.61 (0.28)	0.65 (0.15)
Diseases of the nervous system (G00-G99)	269 (8.4%)	0.57 (0.19)	0.77 (0.17)	0.63 (0.26)	0.65 (0.14)
Diseases of the eye and adnexa (H00-H59)	694 (21.6%)	0.59 (0.17)	0.80 (0.14)	0.67 (0.22)	0.67 (0.12)
Diseases of the ear and mastoid process (H60-H95)	121 (3.8%)	0.58 (0.16)	0.80 (0.16)	0.65 (0.24)	0.67 (0.14)
Diseases of the circulatory system (100-I99)	1625 (50.5%)	0.59 (0.17)	0.80 (0.14)	0.67 (0.21)	0.67 (0.12)
Diseases of the respiratory system (J00-J99)	412 (12.8%)	0.61 (0.17)	0.82 (0.12)	0.69 (0.18)	0.69 (0.10)
Diseases of the digestive system (K00-K93)	1201 (37.3%)	0.59 (0.17)	0.82 (0.13)	0.68 (0.21)	0.68 (0.12)
Diseases of the skin and subcutaneous tissue (L00-L99)	261 (8.1%)	0.61 (0.17)	0.81 (0.13)	0.68 (0.21)	0.68 (0.11)
Diseases of the musculoskeletal system and connective tissue (M00-M99)	2105 (65.5%)	0.60 (0.17)	0.81 (0.13)	0.68 (0.20)	0.68 (0.11)
Diseases of the genitourinary system (N00-N99)	421 (13.1%)	0.61 (0.17)	0.82 (0.14)	0.70 (0.19)	0.69 (0.12)
Other disease	343 (10.7%)	0.57 (0.16)	0.78 (0.16)	0.65 (0.24)	0.66 (0.14)

Note: Chronic diseases were defined with the Korean Standard Classification of Diseases 6th edition (KCD-6). The EQ-VAS values were divided by 100. The EQ-5D general population TTO tariffs were taken from the study by Lee et al. (2009) [26] for South Korea and from the Euroqol group EQ-5D value sets for the UK and Japan [31]

*Abbreviations*: *JP* Japan, *KR* South Korea, *TTO* time trade-off, *VAS* visual analogue scale

The results of regressing patients’ own VAS values (subtracted from 1) on their self-reported health states described in terms of the EQ-5D dimensions and levels are shown in Table 4. The results of the three models, differentiated by level of controls, provided similar results. The largest decrement in all three models were associated with level 3 in the pain/discomfort dimension (coefficient = 0.133, SE = 0.028 in model 3) as well as that in the anxiety/depression dimension (coefficient = 0.129, SE = 0.030 in model 3). However, the adjusted R^2^ was only 0.189, implying that patient VAS values were not well explained by the EQ-5D dimensions and levels across the patients. Notably, similar findings were also observed when including those participants reporting ‘no problems’ in all five EQ-5D dimensions, with the largest decrement associated with level 3 of the pain/discomfort (coefficient = 0.139, SE = 0.027 in model 3) as well as that of the anxiety/depression (coefficient = 0.134, SE = 0.030 in model 3) (adjusted R^2^ = 0.227) (data not shown).

**Table 4:** Coefficients of the EQ-5D dimensions and levels in the patient VAS model

	Model 1 (no controls)	Model 2 (+ control 1)	Model 3 (+ control 2)
Mobility 2	**0.045 (0.007)**	**0.041 (0.007)**	**0.035 (0.007)**
Mobility 3	0.064 (0.036)	0.058 (0.036)	0.062 (0.036)
Self-care 2	**0.033 (0.010)**	**0.031 (0.010)**	**0.030 (0.010)**
Self-care 3	0.048 (0.036)	0.047 (0.036)	0.047 (0.036)
Usual activities 2	**0.036 (0.008)**	**0.035 (0.008)**	**0.030 (0.008)**
Usual activities 3	0.055 (0.033)	0.059 (0.033)	0.047 (0.033)
Pain/discomfort 2	**0.032 (0.008)**	**0.030 (0.008)**	**0.031 (0.008)**
Pain/discomfort 3	**0.137 (0.028)**	**0.134 (0.028)**	**0.133 (0.028)**
Anxiety/depression 2	**0.054 (0.006)**	**0.054 (0.006)**	**0.050 (0.006)**
Anxiety/depression 3	**0.134 (0.030)**	**0.140 (0.030)**	**0.129 (0.030)**
N3	0.020 (0.028)	0.019 (0.028)	0.015 (0.027)
Constant	**0.299 (0.008)**	**0.290 (0.011)**	**0.346 (0.018)**
Adj. R^2^	0.165	0.167	0.189

Note: Values indicate coefficients with standard errors. Those in bold indicate *p*-values< 0.05. Control 1 includes age and sex and control 2 includes age, sex and other variables (marital status, living alone, educational attainment, employment status, income quintile, medical aids and perceived social status). N3 is a dummy variable indicating whether a respondent had level 3 at least in one of the five EQ-5D dimensions

*Abbreviation*: *OLS* ordinary least squares

Table 5 summarizes the relative importance of the EQ-5D five dimensions perceived by patients. Anxiety/depression, on average, had the greatest impact on patient VAS values, followed by pain/discomfort, mobility, self-care and usual activities. This is not consistent with the relative importance perceived by the general population. Mobility appeared to have the greatest impact on the values of health states, followed by usual activities and the rest in the general population model.

**Table 5:** The relative importance of the EQ-5D dimensions – difference between patient and general population valuations

	Patient VAS model			EQ-5D general population TTO model		
	Level 2	Level 3	Mean	Level 2	Level 3	Mean
Mobility	0.994	0.745	0.869	1.758	1.951	1.855
Self-care	0.856	0.558	0.707	0.842	0.635	0.739
Usual activities	0.846	0.561	0.704	0.934	0.971	0.953
Pain/discomfort	0.878	1.596	1.237	0.678	0.705	0.691
Anxiety/depression	1.426	1.541	1.483	0.788	0.738	0.763

Note: The relative importance for each level of each dimension was calculated by dividing the corresponding coefficient from Table 4 (model 3) by the average of the five coefficients at the same level in the same model. For the EQ-5D general population TTO model, the model coefficients were taken from the study by Lee et al. (2009) [26] to calculate the relative importance of the EQ-5D dimensions and levels

*Abbreviations*: *TTO* time trade-off, *VAS* visual analogue scale

**Fig. 1:**
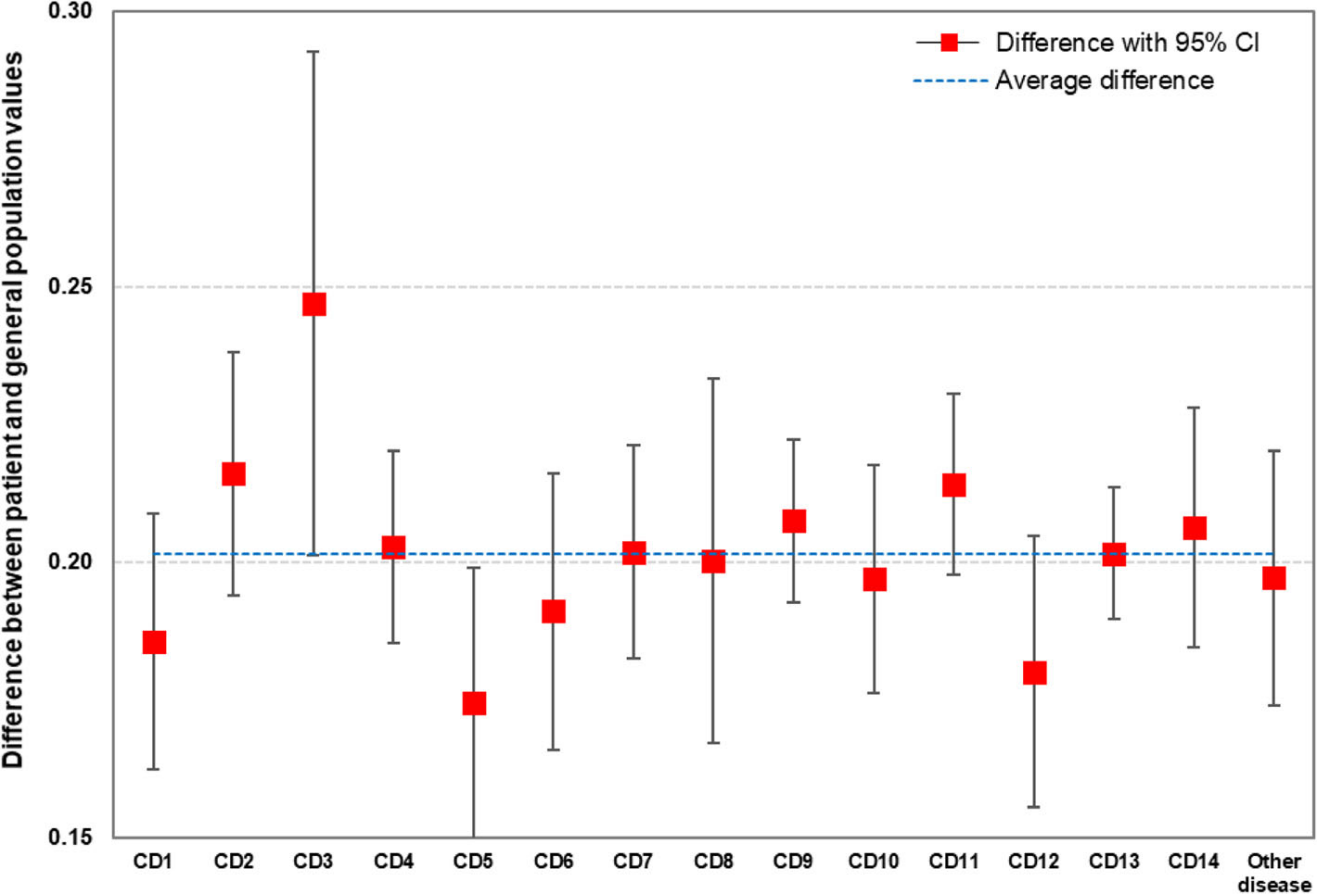
The adjusted mean differences in patient and general population values by type of chronic diseases. CD1, Certain infectious and parasitic diseases; CD2, Neoplasms; CD3, Diseases of the blood and blood-forming organs and certain disorders involving the immune mechanism; CD4, Endocrine, nutritional and metabolic diseases; CD5, Mental and behavioural disorders; CD6, Diseases of the nervous system; CD7,Diseases of the eye and adnexa; CD8, Diseases of the ear and mastoid process; CD9, Diseases of the circulatory system; CD10, Diseases of the respiratory system; CD11, Diseases of the digestive system; CD12, Diseases of the skin and subcutaneous tissue; CD13, Diseases of the musculoskeletal system and connective tissue; CD14, Diseases of the genitourinary system; CI, Confidence interval. Note: These adjusted mean differences reflect the relative differences – not the absolute differences – between patient and general population preferences by type of chronic disease

Figure 1 describes the adjusted mean differences between patient VAS values and general population EQ-5D index scores by each type of chronic diseases, which were measured at the mean levels of socio-demographic variables and at zero values of other chronic diseases (i.e., no other chronic diseases assumed). Notably, these mean differences reflect the relative difference between patient and general population preferences by type of chronic disease. The greatest difference between the two was found in patients with diseases of the blood and blood-forming organs and certain disorders involving the immune mechanism (coefficient = 0.247, 95% CI = 0.201–0.293), followed by those with neoplasms (coefficient = 0.216, 95% CI = 0.194–0.238) and those with diseases of the digestive system (coefficient = 0.214, 95% CI = 0.198–0.231).

## Discussion

The present analysis, using data from a nationally representative South Korean survey, indicates that patients’ valuation of their experienced health states is likely to significantly differ from the general population’s valuation of hypothetical health states. Patients with chronic diseases were found to consider anxiety/depression- and pain/discomfort-related problems more serious than those related to other EQ-5D dimensions such as usual activities and self-care. This fairly contrasts with the preferences of the general population in South Korea [26]. The findings further suggest that the relative difference between patient and general population preferences varies with the type of chronic diseases, which implies that the use of general population preferences could disadvantage certain patient groups because their preferences could be disproportionately reflected in resource allocation decisions.

### Pain/discomfort and anxiety/depression dimensions

Pain/discomfort was the most frequently experienced symptom among patients with chronic diseases – 81.3 and 4.6% reported having ‘moderate problems’ and ‘severe problems’, respectively, in this dimension. More than one third of the patients also reported ‘moderate problems’ in the mobility (40.0%) and the anxiety/depression (35.1%) dimensions, respectively. Problems in self-care and usual activities were relatively less common – the majority (84.9% and 71.2%) reported ‘no problems’ in these dimensions. While pain/discomfort and anxiety/depression were among the most frequently reported symptoms in these patients, the present findings indicate that these are also the dimensions showing the largest discrepancies between patient and general population preferences.

Previous studies have also shown the discrepancies between patient and population values [17], particularly in certain dimensions of health [17, 22, 25, 36–38]. For instance, a recent study [36], involving 282 metastatic breast cancer patients and 333 rheumatoid arthritis patients from Spain, reported the largest disagreements on the anxiety/depression and pain/discomfort dimensions. Although the study examined the differences using the EQ-5D-5 L and TTO values, its findings also suggest that patients consider problems related to anxiety/depression and pain/discomfort more problematic than those related to other health dimensions. The relative importance of anxiety/depression among patients was also reported in other studies [22–24]. For example, Mann et al. [22], using EQ-5D-3 L profiles and VAS values obtained from 3376 UK patients covering eight different conditions, found that patients consider anxiety/depression more problematic, compared to the general population. Similarly, both Burström et al. [23], using experience-based TTO and VAS values from a Swedish sample (*n* = 45,000), and Sun et al. [24], using experience-based VAS values from a Chinese sample (*n* = 120,709), suggest that those individuals experiencing health problems may consider anxiety/depression more problematic than other health problems.

These studies suggest that at least anxiety/depression, and possibly pain/discomfort, could be the least tangible to the general population due to their subjective nature, leading to the largest disagreements on these dimensions. This implies that the impact of these dimensions can only be fully appreciated among the patients who experience those health states. Furthermore, Mann et al. [22] suggested that patients could better adapt to the problems related to mobility, self-care and usual activities, which could make them give smaller weights to these dimensions (i.e., higher values).

There is, however, another study that reported a different pattern of patients’ relative weights given to the EQ-5D dimensions [25]. Using data from the US 2000–2003 Medical Expenditure Panel survey, Rand-Hendriksen et al. [25] compared patient VAS values and general population VAS tariffs. They reported impairments in usual activities to be the most important problems among patients but the least important problems among the general population.

Contrary to the aforementioned studies, however, Rand-Hendriksen et al. [25] argued that impairments in usual activities could be the most difficult health problems for the US general public to imagine. Given this mixed evidence/argument, it is not clear as to which dimensions are relatively less tangible to the general public who have not experienced those health states. It may however be possible that the type of tangible dimensions to the general public and the extent of patient adaptation differ across countries. Patients could better adapt to usual activities or self-care problems in the countries where strong social support is available for those affected. On the contrary, the general public could overvalue usual activities or self-care activities in the countries where there is no adequate social protection system. Notably, South Korea’s public social spending accounts for 10.4% of its GDP, which is only half the OECD average of 21.0% in 2016 [39]. In addition, South Koreans work the second-longest hours among OECD countries [40]. It is possible that an individual’s functioning can be considered particularly important in such environments – this aspect could have been reflected in the general population’s valuation in South Korea. Furthermore, the general public could have more difficulties to imagine and appreciate those hypothetical health states involving severe pain/discomfort and/or anxiety/depression problems in the countries where people are less inclined to verbalize and seek help for their physical and/or mental health problems. In relation to this, a recent review by Steel et al. confirms consistently lower prevalence estimates for common mental disorders in Asia than in European or English-speaking countries [41], whereas OECD statistics indicate that the suicide rate of Asian countries is far above the OECD average of 12.0 in 2013 [42]. South Korea in particular revealed the highest suicide rate with 29.1 deaths per 100,000 population. This may imply that recognition of mental disorders or help-seeking for mental health problems is particularly low in Asian countries, which may affect the general population’s valuation for these health dimensions.

The present study also shows that the relative difference of the discrepancy between patient and general population preferences varies across the types of chronic diseases. The largest discrepancy, in a relative sense, was found in diseases of the blood and blood-forming organs and certain disorders involving the immune mechanism, followed by neoplasm and diseases of the digestive system. However, it seems that the discrepancy becomes apparent only when patients have severe health problems. For instance, mental and behavioural problems exhibited the least discrepancy between patient and general population values, which was rather unexpected. This was likely because the percentage of health states involving severe health problems was very low in this KHP survey data. Notably, patients with mental and behavioural problems described their health state most frequently as 11121 (14.6%), followed by 11112 (13.9%), 11122 (12.4%) and 22222 (9.4%).

Taken together, these findings indicate that the use of general population tariff, as recommended by the South Korean pharmaco-economic evaluation guideline [8], could give somewhat lower priority to those treatments that improve severe pain/discomfort and anxiety/depression problems, compared to the use of patient values in South Korea. Although further work is clearly needed to investigate the source of the discrepancy between patient and general population preferences within and across countries, these findings raise concern over the sole use of general population preferences in resource allocation decision making, especially for those treatments that aim to alleviate severe pain/discomfort and/or anxiety/depression symptoms.

### Limitations

This study has several limitations that should be taken into account when interpreting the results. Firstly, this study did not achieve the full comparability between patient and general population values. While patient values were derived with the VAS, general population values were elicited with the TTO method. Available evidence suggests that different valuation techniques produce different utility weights [43]. It should be noted, however, that both TTO and VAS measures have been widely used in QoL research. If they provide different patterns on the relative importance – not absolute importance – of the EQ-5D five dimensions, their use, especially the VAS, should be reconsidered in QoL research. In addition, previous studies, comparing patient VAS values with general population VAS tariffs, inherently assume that any differences between the two, to some extent, translate into differences in choice-based valuations such as the TTO. Otherwise, those empirical findings cannot provide meaningful policy implications because choice-based tariffs are dominantly used in formal economic evaluations for healthcare decision-making. The present study should nevertheless be taken as the first step to provide some early evidence on the areas of discrepancies between patient and general population preferences in South Korea. Further research, using the same valuation techniques, is clearly warranted to confirm the present findings.

Secondly, despite a wide use of the VAS, it should be acknowledged that the VAS is likely more prone to measurement errors compared to other valuation techniques [44]. Notably, the present patient VAS model had the adjusted R^2^ of 0.189 (and R^2^ of 0.20), which is much lower than that for the general population TTO model (0.984 with the N3 model) reported in the study by Lee et al. [26]. EuroQol population-based valuation studies, however, reported R^2^ of the VAS models (ranging from 0.47 for the UK to 0.97 for Spain) at least similar to that of the TTO models (ranging from 0.38 for the Netherlands to 0.66 for Denmark) [31]. In addition, Burström et al. [23], using Swedish experience-based VAS and TTO values, reported a greater adjusted R^2^ for the VAS N3 model (0.4875) than that for the TTO N3 model (0.2393). This indicates that the low level of the adjusted R^2^ in this patient model cannot be attributed to the use of the VAS. Instead, it could to some extent be due to the burden placed on the KHP respondents – a great number of questions administered could have increased response burden and subsequently lowered response accuracy. However, there is also a Dutch study that reported a low level of R^2^ (0.22), using data from a randomized controlled trial, of which response accuracy is likely better than that of a large-scale survey [45]. This could in part indicate that the EQ-5D-3 L descriptive system does not comprehensively capture patients’ HRQoL, as suggested by recent studies [46, 47]. For instance, using the EQ-5D-3 L and the WHOQOL-BREF (World Health Organization Quality of Life Scale – Abbreviated form) [48], Jelsma et al. [46] reported that the addition of domains such as concentration and sleep may improve the explanatory power to predict VAS values. In addition, Pietersma et al. [47] conducted a three-stage online Delphi consensus procedure to identify the key domains of HRQoL and found that ‘self-acceptance’, ‘self-esteem’ and ‘good social contacts’ may be perceived as essential both in patients and non-patents, which are not captured in the EQ-5D-3 L description. Further research is warranted to explain which additional domains are most suitable to comprehensively reflect HRQoL.

Finally, consistent with Burström et al. [23], rescaling, which makes the VAS values to be anchored between ‘dead’ and ‘perfect health’ and therefore makes the values better comparable to the general population tariffs, was not done because the VAS value for ‘dead’, drawn from a representative sample of South Korean patients, were not available. Given these limitations, it is possible to make a direct comparison only between the relative weights – not the absolute weights - of the EQ-5D dimensions given by patients and the general population.

## Conclusions

Despite these limitations, this study provides a glimpse of what dimensions of health patients with chronic diseases in South Korea consider more problematic. These findings further confirm the differences in the relative importance of the EQ-5D dimensions between patients and the general population. Compared to the general population, South Korean patients may consider pain/discomfort and anxiety/depression more problematic than impairments in mobility, self-care or usual activities, especially when the symptoms are severe. The findings also indicate that the extent of the discrepancies between patient and general population values, in a relative sense, vary across the types of chronic diseases. Although this study cannot address the question of whose values should be used in economic evaluations, which is ultimately a normative question, these findings suggest that both patient and general population preferences be reflected in resource allocation decisions at least for those treatments that aim to alleviate severe pain/discomfort and/or anxiety/depression symptoms. Further research is, nevertheless, warranted to validate these findings using the same valuation techniques for both patients and the general population.

## Abbreviations

AD: Anxiety and Depression
GDP: Gross Domestic Product
HIRA: Health Insurance Review and Assessment services
HRQoL: Health-Related Quality of Life
ICER: Incremental Cost-Effectiveness Ratio
KCD-6: Korean Standard Classification of Diseases 6th edition
KHP: Korea Health Panel
MO: Mobility
OECD: Organization for Economic Co-operation and Development
OLS: Ordinary Least Squares
PD: Pain and Discomfort
QALY: Quality-Adjusted Life-Year
QoL: Quality of Life
SC: Self-care
SD: Standard Deviation
SE: Standard Error
TTO: Time Trade-off
UA: Usual activities
VAS: Visual Analogue Scale
